# A holobiont approach towards polysaccharide degradation by the highly compartmentalised gut system of the soil-feeding higher termite *Labiotermes labralis*

**DOI:** 10.1186/s12864-023-09224-5

**Published:** 2023-03-15

**Authors:** Martyna Marynowska, David Sillam-Dussès, Boris Untereiner, Dominika Klimek, Xavier Goux, Piotr Gawron, Yves Roisin, Philippe Delfosse, Magdalena Calusinska

**Affiliations:** 1 Environmental Research and Innovation Department, Luxembourg Institute of Science and Technology, 41 Rue du Brill, L-4422 Belvaux, Luxembourg; 2 Evolutionary Biology and Ecology, Université Libre de Bruxelles, 50 Avenue F.D. Roosevelt, B-1050 Brussels, Belgium; 3 University Sorbonne Paris Nord, Laboratory of Experimental and Comparative Ethology, LEEC, UR 4443, F-93430 Villetaneuse, France; 4 Luxembourg Centre for Systems Biomedicine, University of Luxembourg, 6 Avenue du Swing, L-4367 Belvaux, Luxembourg; 5 Vice-Rectorate for Research, University of Luxembourg, 2 Avenue Des Hauts-Fourneaux, L-4365 Esch-Sur-Alzette, Luxembourg

**Keywords:** Carbohydrate active enzyme, Higher termite gut system, Lignocellulose, *Labiotermes labralis*, Metagenomics, Metatranscriptomics, Polysaccharide degradation

## Abstract

**Background:**

Termites are among the most successful insects on Earth and can feed on a broad range of organic matter at various stages of decomposition. The termite gut system is often referred to as a micro-reactor and is a complex structure consisting of several components. It includes the host, its gut microbiome and fungal gardens, in the case of fungi-growing higher termites. The digestive tract of soil-feeding higher termites is characterised by radial and axial gradients of physicochemical parameters (*e.g.* pH, O_2_ and H_2_ partial pressure), and also differs in the density and structure of residing microbial communities. Although soil-feeding termites account for 60% of the known termite species, their biomass degradation strategies are far less known compared to their wood-feeding counterparts.

**Results:**

In this work, we applied an integrative multi-omics approach for the first time at the holobiont level to study the highly compartmentalised gut system of the soil-feeding higher termite *Labiotermes labralis*. We relied on 16S rRNA gene community profiling, metagenomics and (meta)transcriptomics to uncover the distribution of functional roles, in particular those related to carbohydrate hydrolysis, across different gut compartments and among the members of the bacterial community and the host itself. We showed that the *Labiotermes* gut was dominated by members of the Firmicutes phylum, whose abundance gradually decreased towards the posterior segments of the hindgut, in favour of Bacteroidetes, Proteobacteria and Verrucomicrobia. Contrary to expectations, we observed that *L. labralis* gut microbes expressed a high diversity of carbohydrate active enzymes involved in cellulose and hemicelluloses degradation, making the soil-feeding termite gut a unique reservoir of lignocellulolytic enzymes with considerable biotechnological potential. We also evidenced that the host cellulases have different phylogenetic origins and structures, which is possibly translated into their different specificities towards cellulose. From an ecological perspective, we could speculate that the capacity to feed on distinct polymorphs of cellulose retained in soil might have enabled this termite species to widely colonise the different habitats of the Amazon basin.

**Conclusions:**

Our study provides interesting insights into the distribution of the hydrolytic potential of the highly compartmentalised higher termite gut. The large number of expressed enzymes targeting the different lignocellulose components make the *Labiotermes* worker gut a relevant lignocellulose-valorising model to mimic by biomass conversion industries.

**Supplementary Information:**

The online version contains supplementary material available at 10.1186/s12864-023-09224-5.

## Background

Termites are among the most important lignocellulose (plant biomass) decomposing organisms on Earth [1]. Over evolutionary time, they have developed essential microbial associations, enhancing their metabolic capabilities and allowing them to feed on a broad range of dietary substrates. While the evolutionarily basal “lower termites” live in symbiosis with gut flagellates and prokaryotes, “higher termites” (Termitidae family) possess an entirely prokaryotic gut community [1]. The feeding regime of the former group is generally restricted to wood, while the latter has diversified its food habits to thrive on plant biomass over a wide range of the humification gradient, including wood at various stages of decomposition, leaf litter, humus and soil [2]. A higher taxonomic diversity of termites lies towards the humified end (*i.e.* soil), and in tropical ecosystems, soil-feeding termites account for 60% of the known termite species [3].

Early studies of biomass digestion by soil-feeding termites provided little evidence for a microbe-assisted lignocellulosic polysaccharide degradation, as no cellulolytic organisms were isolated from the soil-feeding termite guts, and overall cellulolytic enzyme activities were weak (summarised in [4]). Later, the soil-dwelling fauna together with its gut microbiota was evidenced to decompose soil organic matter more quickly than initially recognised [5] and cellulose mineralisation was confirmed for the soil-feeding termite *Cubitermes orthognathus* [6]. More recent studies have taken the advantage of high-throughput sequencing approaches to offer deeper insights into the metabolic potential of the gut prokaryotic communities along a wood-soil decomposition gradient [7–10]. In line with the feeding habits of soil feeders, which rely on the nitrogen-rich fraction of soil including bacterial biomass [11], the diversity of lignocellulose-targeting carbohydrate-active enzymes (CAZymes) encoded in gut microbial genomes appears to be considerably lower than for wood-feeding termites [7]. Contrary to this, a metatranscriptomic approach applied to the whole gut extracts of different higher termite species, showed that the diversity of expressed CAZyme genes and in particular glycoside hydrolases (GH) was comparable between termites representing different feeding guilds [9]. Moreover, a higher number of unique bacterial GHs was characterised in soil feeders and on average a higher diversity of genes was assigned per GH family. The metatranscriptomic abundance of endoglucanase gene transcripts (*i.e.* cellulose-targeting enzymes) was comparable between the CAZyomes (*i.e.* CAZyme repertoire) of soil and plans fibre feeders, while endoxylanase-encoding gene transcripts clearly dominated the CAZyomes of soil-feeding termites [9]. The exploration of the soil-feeding termite CAZyome may thus reveal new enzymes that decompose difficult to access soil lignocellulosic components efficiently. Indeed, the soil matrix is enriched with recalcitrant materials, such as lignin and other aromatics, which aggregate with polysaccharides, limiting their accessibility to the enzymatic machinery [12].

**Fig. 1:**
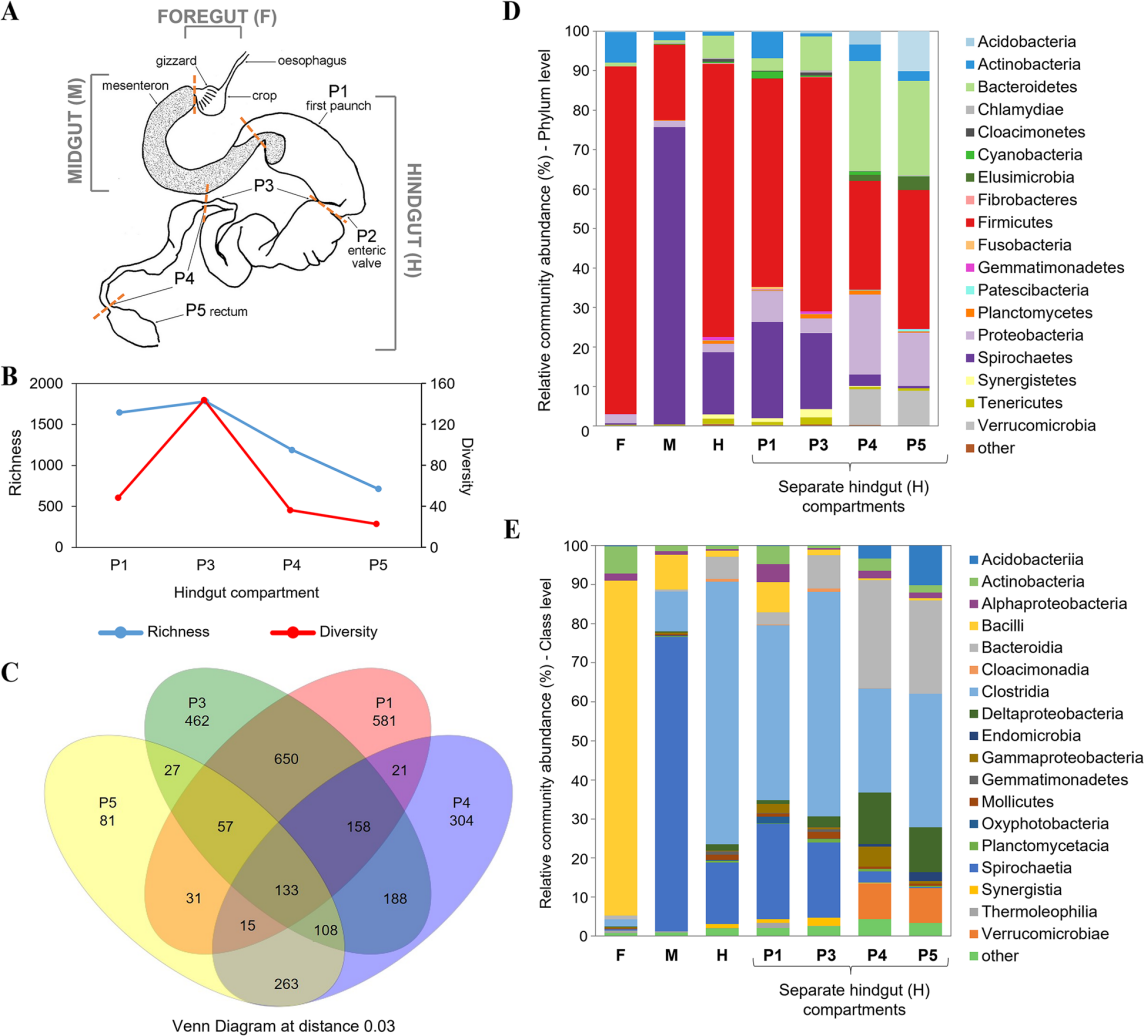
Structure of the symbiotic community in the major gut compartments of *L. labralis* analysed by 16S rRNA gene amplicon high-throughput sequencing. **A** Schematic of the *L. labralis* digestive tract with the site of the cutting between the different compartments indicated with a dashed line. **B** Richness (number of observed OTUs inferred using a sobs calculator) and diversity (inferred using the inverse of the Simpson diversity estimator) of bacterial communities in the different hindgut compartments. **C** Venn diagram of shared and unique OTUs in the different sections of the hindgut (P1-P5). Relative community abundance in different gut sections at the phylum (**D**) and class (**E**) levels

*Labiotermes labralis* (*L. labralis*; Termitidae, Syntermitinae) is one of the most abundant soil feeders in the Amazonian primary forest [13], playing a fundamental role in soil structure and fertility [14]. It represents the feeding group IV, referred to as “true soil feeders”, relying on cellulosic and non-cellulosic polysaccharides in an extremely amorphous and refractory form [2]. The digestive tract of an *L. labralis* worker is highly compartmentalised, which is a characteristic of all soil feeders, and it includes the foregut (F, crop and gizzard), the midgut (M, mesenteron) and the hindgut (H), which is further differentiated into P1 (paunch), P2 (enteric valve), dilated P3 and P4 (colon), as well as P5 (rectum; Fig. 1A) [15]. The intestine of soil feeders is longer and the distinct gut segments are characterised by axial and radial gradients of various physicochemical parameters, including pH, O_2_ and H_2_ partial pressure [15, 16]. The increase in length, volume and compartmentalisation has transformed the soil-feeding termite gut, and especially its hindgut, into a series of complex microbial habitats densely colonised by distinct populations [17]. Notably, the anterior hindgut (especially the P1 compartment) is characterised by an unusually high pH, equal to the highest values ever encountered in biological systems [18], bringing attention to the bacterial communities living in such extreme conditions and the enzymes that they employ. Currently, the polysaccharide degradation strategies of the termite gut system with a dietary preference for soil remains largely uncharacterised compared to their wood-feeding counterparts.

In this study, we investigated the highly compartmentalised gut system of the soil-feeding termite,* L. labralis* for the first time at the holobiont level, using an integrative multi-omics approach applied to both the termite and its gut microbiome. First, using 16S rRNA gene amplicon profiling, we confirmed that the major gut compartments differ in the diversity and composition of the bacterial communities that they harbour. Next, by applying de novo metagenomics and further contigs binning, we were able to reconstruct novel bacterial metagenome assembled genomes (MAGs) and link the presumed lignocellulolytic activities in the different gut sections to specific community members. Subsequently, by using the de novo metatranscriptomics and host transcriptomics, we established a gut reference catalogue of CAZymes together with their expression profiles across the different gut compartments. This allowed us to further characterise host and microbial strategies associated with the decomposition of the different lignocellulose fractions, and other biodegradable components of the termite diet, including non-cellulosic polysaccharides. Contrary to expectations based on previous studies*,* we showed that *L. labralis* gut microbes express a high diversity of CAZymes involved in cellulose and hemicellulose degradation, making the soil-feeding termite gut a unique reservoir of lignocellulolytic enzymes with considerable biotechnological interest. Finally, we evidenced that the host cellulase paralogs have different structures and phylogenetic origins, which possibly allows the termite to feed on distinct polymorphs of cellulose retained in soil.

## Methods

### Sampling and extraction of nucleic acids

A nest of the soil-feeding higher termite *Labiotermes labralis* was identified in the tropical forest near Petit Saut in French Guiana in April 2018 and mature workers were collected. The species’ taxonomic designation was primarily assigned by morphological features, and was further confirmed by the partial sequencing of the cytochrome oxidase subunit 2 gene, as previously described [9]. The nucleotide sequence is available in GenBank under accession number MN803316. Mature workers retrieved from the nest were cold-immobilised, surface-cleaned with 80% ethanol and 1 × PBS and decapitated. The guts of 30 workers were immediately dissected and separated into individual compartments, including foregut and midgut, as well as the different sub-sections of the hindgut (P1, P3, P4, P5; Fig. 1A). The mixed segment was not collected separately, and the separation of the midgut from P1 was done as illustrated in Fig. 1A. Homologous gut sections were pooled and preserved in RNAlater solution (ThermoFisher Scientific) at 4 °C until further processing. DNA and RNA were co-extracted from the different gut sections using the AllPrep PowerViral DNA/RNA Kit (Qiagen), as described previously [19]. An additional bead-beating step with 0.1 mm glass beads at 20 Hz during 2 min was introduced to optimise the bacterial cell wall lysis. Following the extraction of the macromolecules, the eluent was divided into two parts and treated with 1 μL of 10 μg/ml RNaseA (Sigma) for 30 min at room temperature, and TURBO DNase (Invitrogen) according to the manufacturer´s instructions, respectively, to obtain pure DNA and RNA fractions. The quality and quantity of extracted nucleic acids was assessed with the Bioanalyser (Agilent) and Qubit (Invitrogen), respectively. DNA and RNA extract were stored at -20 °C and -80 °C, respectively, until further library preparations.

### 16S rRNA gene amplicon high-throughput sequencing and data analysis

The bacterial 16S rRNA gene amplicon libraries were prepared for the different gut sections and sequenced using the Illumina MiSeq approach as previously described [19]. Briefly, a modified version of universal primers S-D-Bact-0909-a-S-18 and S-*-Univ-*-1392-a-A-15 [20] and Nextera XT Index Kit V2 (Illumina) were used together with Q5 Hot Start High-Fidelity 2 × Master Mix (New England Biolabs) in a two-step PCR reaction to amplify a fragment of around 484 bp, spanning the V6–V8 region [21]. Following sequencing, Usearch v.7.0.1090_win64 software [22] was used for the quality trimming (fastq-maxee 1, fastq_minlen 400), chimera check, removal of singletons and assignment of sequences to operational taxonomic units (OTUs) at the 97% similarity level, according to the pipeline described previously [9]. The taxonomic affiliation of the resulting OTUs was performed with the SILVA database v.132 [23] using mothur [24] (Additional file 1: Table S1). The sequencing reads are available in the Sequence Read Archive (SRA) database under accession numbers SAMN14943942, SAMN14943943, SAMN14943945, SAMN14943946, SAMN14943947 and SAMN14943948. Bacterial community richness and diversity were calculated on the normalised reads of bacterial origin using sobs and invsimpson calculators, respectively, implemented in mothur [24].

### Metagenomics and assembly of bacterial genomes

A DNA sample representative of the hindgut luminal fluid was used for metagenomic sequencing in order to reconstruct genomes and larger genomic fragments of the dominant microbes. Although care was taken to avoid the termite gut tissue during the material sampling, a NEBNext Microbiome DNA Enrichment Kit (NewEngland BioLabs) was used to enrich the fraction of prokaryotic DNA from the hindgut luminal fluid DNA extract. A library was prepared using the Nextera XT DNA Library Preparation Kit (Illumina), and was quantified with the High Sensitivity DNA Kit (Agilent) and KAPA SYBR FAST Universal qPCR Kit and sequenced using the Illumina NextSeq 500/550 Mid Output v2-300 Kit. Sequencing was done at the University of Luxembourg. The sequencing reads are available in the SRA database under accession number SAMN14943944. Raw reads were quality trimmed in CLC Genomics Workbench v.9.5.2, using a Phred quality score of 20, minimum length of 50, removal of 3 nt at 5’ end and allowing no ambiguous nucleotides. Quality-trimmed reads were assembled using the CLC’s de novo assembly algorithm in a mapping mode, using automatic bubble size and word size, a minimum contig length of 1000, mismatch cost of 2, insertion cost of 3, deletion cost of 3, length fraction of 0.9, and similarity fraction of 0.95. Sequencing details are given in Table 1. Initial MetaBAT2 binning [25] resulted in a total of 143 reconstructed MAGs (Additional file 1: Table S2). Subsequently, a MetaWRAP pipeline [26] was used to refine the resulting MAGs to the species level, resulting in 39 good-quality MAGs (Additional file 1: Table S3). The completeness and contamination of the generated MAGs were assessed with checkM [27]. The taxonomic affiliation of the MAGs was assessed using the GTDB-Tk tool [28]. The novelty of the resulting MAGs was assessed with FastANI [29], using the previously generated database of good-quality MAGs from the termite gut system [30] (Additional file 1: Table S4). The complete results on the good-quality MAGs are reported in Additional file 1: Table S3. Metagenomic assemblies as well as the reconstructed- and partially reconstructed MAGS are available upon request. Standard deviation from the calculated mean is indicated as “ ± ” throughout the manuscript.

**Table 1:** Data summary of metagenomics, metatranscriptomics and host transcriptomics

	De novo (meta)transcriptomics						MG
	Foregut	Midgut	Hindgut				
			P1	P3	P4	P5	LF
Termite host							
Total quality-trimmed reads (Million)	21.4	29.3	26.9	23.8	27.3	20.6	NA
Co-assembly length	94,526,795						NA
Number of contigs	218,010						NA
Number of ORFs	124,142 (14,281 of Insecta origin)						NA
Number of CAZymes	313 (184 of Insecta origin)						NA
Gut microbiome							
Total quality-trimmed reads (Million)	65.5	71.7	66.3	67.1	55	64.3	270,5
Co-assembly length (bp)	790,796,077						642,017,023
Number of contigs	2,090,052						304,288
Number of ORFs	2,114,234 (369,503 of prokaryotic origin)						763,653
Number of CAZymes^a^ (assigned to MAGs)	3534 (197)						1713
Contigs binned to MAGs (% MG reads)	NA						11,098 (8.6%)
% of mRNA reads mapped to good-quality MAGs	2.99	5.22	3.79	8.81	4.7	3.2	NA

*MG* Metagenomics, *LF* Luminal fluids, *NA* Not applied

^a^Only CAZymes taxonomically assigned to Prokaryota were considered in this analysis

### Metatranscriptomics and host transcriptomics

The previously optimised in-house metatranscriptomics framework [19], based on the combination of the Ribo-Zero Gold rRNA Removal Kit “Epidemiology” (Illumina) and the Poly(A)Purist MAG Kit (Ambion) was used to enrich prokaryotic mRNA and termite mRNA, respectively, from the total RNA extracted from six different gut sections (foregut, midgut, P1, P3, P4 and P5). A detailed procedure of prokaryotic mRNA enrichment was described in our previous study (“Pipeline RZ” in [19]). The enrichment of host mRNA was performed according to the same pipeline, however at the step the Poly(A)Purist MAG (Invitrogen) was used, Oligo(dT) MagBeads with bound poly-A-mRNA were retained and further separate libraries were constructed for the different gut sections, according to the manufacturer’s protocol. A SMARTer Stranded RNA-Seq Kit (TakaraBio) was used according to the manufacturer’s instructions in order to prepare metatranscriptomic libraries, using the enriched prokaryotic and host mRNA as input. The resulting libraries were quantified with a High Sensitivity DNA Kit (Agilent) and KAPA SYBR FAST Universal qPCR Kit. Six prokaryotic metatranscriptomic libraries were pooled and six host transcriptomic libraries were pooled and sequenced using an Illumina NextSeq 500/550 High Output v2-300 Kit. The sequencing reads are available in the SRA database under the biosample accession numbers given above. Raw reads were quality trimmed in CLC Genomics Workbench v.9.5.2, using a Phred quality score of 20, minimum length of 50, removal of 3 nt at 5’ end and allowing no ambiguous nucleotides. Contaminating rRNA reads were further removed using SortMeRNA 2.0 software [31]. The non-rRNA reads were used to perform two separate de novo (meta)transcriptomic co-assemblies, for the microbiome and host, respectively, using the CLC’s assembly algorithm in mapping mode with default parameters, except for the minimum contig length of 200, length fraction of 0.90 and similarity fraction 0.95. To assess the relative abundances of transcripts across the samples studied, for both the de novo assembled metatranscriptome and the host transcriptome, sequencing reads were mapped back to the annotated transcript sets using the CLC “RNA-seq analysis” mode, with default parameters, except for the minimum similarity of 0.95 over 0.90 of the read length, the specificity of both strands and a maximum one of hit per read. The mapping results were represented as transcripts per million (TPMs), which resulted directly in normalised reads counts. Additionally, the trimmed and filtered metatranscriptomic reads for the prokaryotic part were also mapped to the metagenomic reconstructions in order to determine the activity of MAGs in the respective gut compartments, and more specifically to assess the gene expression levels of CAZymes encoded in the different MAGs. Transcripts of eukaryotic origin taxonomically assigned to Insecta were further evaluated for the completeness of the de novo reconstructed transcriptome with the BUSCO pipeline [32] using the Arthropoda database (odb10). Metatranscriptomics assembly and reconstructed termite host transcriptome are available upon request.

### Functional annotation and data analysis

Open reading frames (ORFs) were predicted using Prodigal [33] for the prokaryotic part, and GeneMark [34] for eukaryotic genes. Taxonomic annotation of the resulting genes (metagenomics) and gene transcripts (metatranscriptomics) was performed using kraken2 software [35] using the NCBI non-redundant nucleotide database. Gene transcripts that matched with the MAGs reconstructed in this study and elsewhere [30] were further re-annotated taxonomically. Functional annotation was done with DIAMOND [36] using the NCBI non-redundant protein database. The functional assignment to the Kyoto Encyclopedia of Genes and Genomes (KEGG) orthologous categories (KOs) was done with GhostKoala [37]. Metabolic pathways and modules were reconstructed using the tool integrated in the online version of the KEGG database [38]. CAZymes coding genes were searched with dbCAN2 [39] on the CAZy database v6 [40], and positive hmm search hits with an e-value of < 10^–18^ threshold and coverage of > 0.35 were retained for further analysis. CAZyme clusters were detected using the CGC_Finder.py script implemented in the dbCAN2 [39]. Phylogenetic analyses were performed on MAFFT-aligned protein sequences [41] using MEGA software [42].

## Results and discussion

### Structure of the termite gut microbiome in the different gut compartments

The composition of microbial communities residing in the main *L. labralis* gut compartments was first assessed with 16S rRNA gene amplicon high-throughput sequencing. For this purpose, DNA was extracted from individually sampled gut sections, including the foregut, midgut and four major sections of the hindgut (P1, P3, P4 and P5; Fig. 1A). The sequencing resulted in 298,649 quality-trimmed reads, which were further assigned to 3,478 bacterial OTUs (Additional file 1: Table S1). Bacterial richness and diversity (based on normalised read counts to 27.5 k reads) were the highest in the P3 section of the hindgut and dropped towards posterior segments of the gut (Fig. 1B, C). The number of reads generated for the foregut and midgut samples was significantly lower, therefore the diversity indices were not calculated for these compartments. Following the taxonomic annotation, in total, 26 bacterial phyla were identified in the different gut compartments, with the highest number of OTUs being associated with Firmicutes, followed by Proteobacteria, Bacteroidetes and Spirochaetes. Firmicutes was the most abundant in the foregut and hindgut sections, with Bacilli being the dominant class in the foregut, and Clostridia in the hindgut compartments (Fig. 1D, E). The midgut seemed mainly colonised by Spirochaetes, which were also abundant in the anterior hindgut, however different OTUs dominated in the different gut compartments (Additional file 1: Table S1), showing the niche preference of the different strains. At the OTU level, despite an overlap in species between the different hindgut sections (Fig. 1C), a significant dominance of single OTUs was noticeable in the different gut compartments. For example, *Treponema* OTU_2 accounted for 13% of the bacterial community in P1, while it was nearly absent in P2-P5 (less than 0.05%). Similarly, Ruminococcaceae OTU_13 (Firmicutes) dominated in P3 (4.2%), Desulfobulbaceae OTU_4 (Proteobacteria) in P4 (10%) and Dysgonomonadaceae OTU_8 (Bacteroidetes) in P5 (14.6%; Additional file 1: Table S1). Interestingly, one OTU dominated the Verrucomicrobia community in the posterior hindgut compartments (accounting for 7.9% of bacterial community) and was associated with *Diplosphaera* genus (Additional file 1: Table S1). We did not manage to get a good-quality MAGs of this species (see below) to further reconstruct its metabolic potential. Nor was it reconstructed in a recent study of termite gut MAGs [30]. However, a previously characterised *Diplosphaera colitermitum*, isolated from the lower termite *Reticulitermes flavipes*, indicated a potential to degrade cellulose, even though the in vitro substrate utilisation spectrum was limited to starch and a few mono- and disaccharides [43]. Microenvironmental heterogeneity, including the difference in the oxygen gradient and pH across the termite gut, is the main driving force behind the dominance of the different bacterial species in separate gut sections [17]. Although these parameters have not yet been specifically assessed for *L. labralis*, to date there is a clear trend in all soil-feeding higher termites, which might be extrapolated to our study [15, 44]. In general, the foregut and midgut parts are neutral and characterised with the highest oxygen partial pressure, while the anterior hindgut compartments, and particularly P1, are highly alkaline and anaerobic.

No analysis of microbial communities separately in the different gut compartments of *Labiotermes* species has been done previously. However, analogous studies performed for other soil-feeding termites show similar trends, where the dominance of Firmicutes is most prominent in the anterior hindgut compartments, while Bacteroidetes and Proteobacteria become more abundant towards the end of the termite gut tract [17, 45]. To allow for a further comparison with the previously studied *Labiotermes* spp., we also analysed the average community composition of the hindgut by combining reads from the different hindgut sections together and averaging the abundance of specific microbial groups in a pool. As a result, the clear dominance of Firmicutes and to a lower extent Spirochaetes showed a strong resemblance to a previously published record [9]. Nevertheless, small differences in niche preferences of certain species can only be highlighted when analysing the different gut sections separately. Notably, the decreasing abundance of Firmicutes in the posterior hindgut in favour of Bacteroidetes, Proteobacteria and Verrucomicrobia, could not be deduced solely from the combined hindgut analysis.

### Hindgut metagenomics and reconstruction of metagenome assembled genomes

To further assess the ability of specific bacteria to digest carbohydrates in the termite tract, we separately extracted the hindgut luminal fluid combining all the hindgut compartments and sequenced the whole community DNA. The sequencing effort resulted in an assembly of 304,288 contigs harbouring 763,653 genes. We initially reconstructed 143 microbial MAGs of different completeness and contamination (Additional file 1: Table S2). They were further refined, resulting in 39 good-quality microbial MAGs, of which 38 were classified as being of bacterial origin (Additional file 1: Table S3). The final collection of good-quality MAGs, with an average completeness of 71.4% ± 13.3 and contamination of 2.7% ± 2.4 was used for all comparative analyses. The majority of MAGs were assigned to Firmicutes, mainly Clostridia, which correlated with the dominance of these bacteria in the different hindgut compartments of *Labiotermes* workers (Fig. 1E). No Bacilli MAG was reconstructed, showing that species present in the foregut compartment were unlikely to migrate to the hindgut, possibly due to very different pH preferences. Only a few MAGs were assigned to Fibrobacteres, Proteobacteria and Spirochaetes, and no good-quality Bacteroidetes MAGs were recovered here, despite Bacteroidetes being the second most abundant bacterial phylum in P4 and P5 hindgut sections, based on the community structure analysis (Fig. 1D). We further compared our draft genomes to the previously published collection of MAGs from the gut of different higher termites, including 169 MAGs from the *Labiotermes* sp. gut [30]. Roughly 11 MAGs showed average nucleotide identity (ANI) exceeding 70%, with an average of 78.7% ± 2.4. None of the MAGs exceeded the species-level threshold of 95% ANI, suggesting that all the good-quality MAGs reconstructed in our study represented novel bacterial species (Additional file 1: Table S4).

**Fig. 2:**
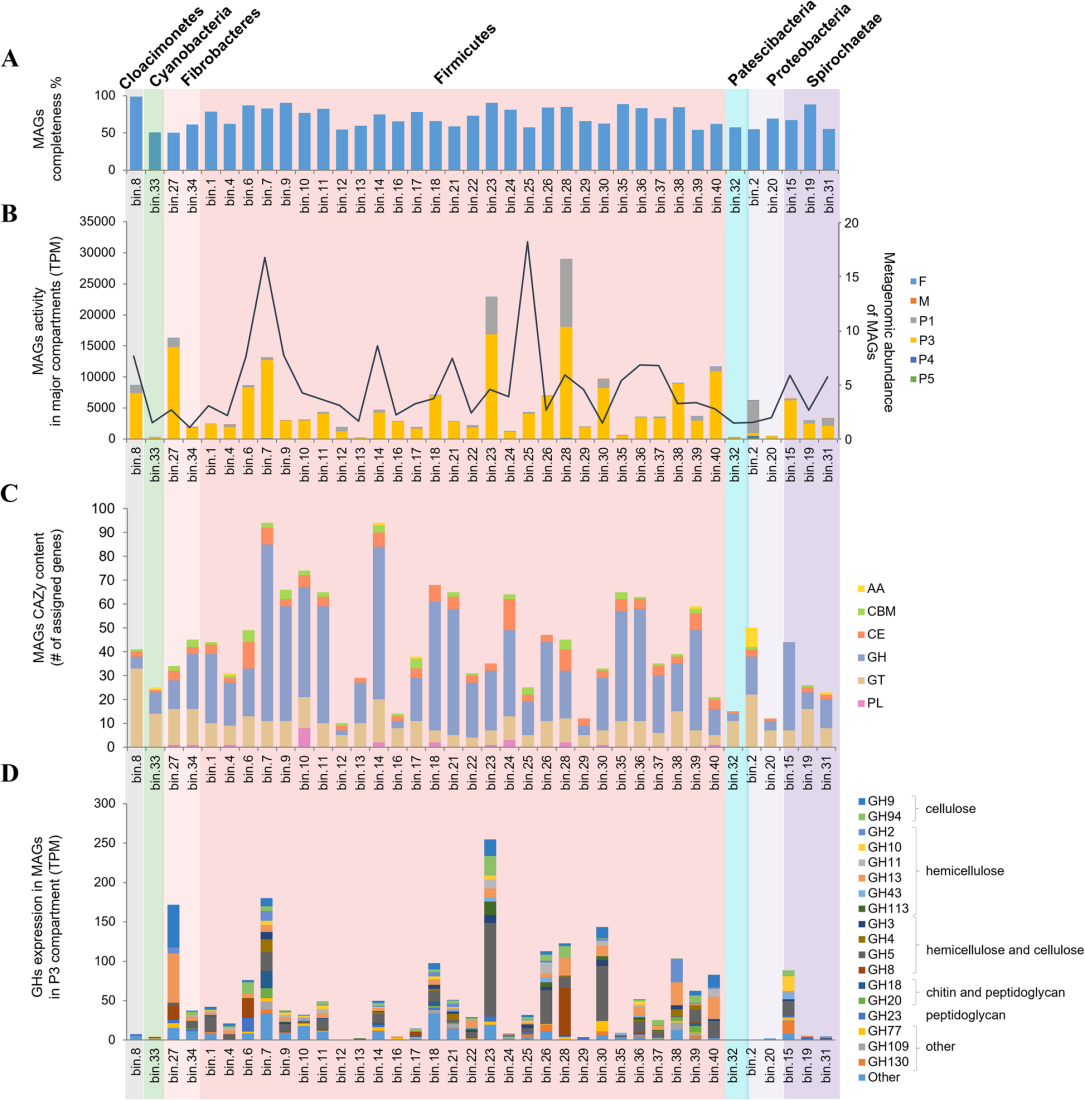
Characterisation of reconstructed bacterial metagenome assembled genomes (MAGs). **A** MAG completeness (%). **B** Metagenomic abundance (indicated with a solid line) and activity of MAGs in the different gut compartments. **C** Carbohydrate active enzyme (CAZyme) content of the reconstructed MAGs. **D** Expression of the dominant GHs present in the reconstructed MAGs in the P1 and P3 compartments

Single MAGs of rare bacterial phyla in the context of the termite gut included Cloacimonetes (MAG8; Fig. 2), Cyanobacteria (MAG33) and Patescibacteria (MAG32). The former was among the most metagenomically abundant bacterial MAGs (Fig. 2B), while the latter two were less abundant, and only characterised with less well reconstructed genomes. Cloacimonetes bacteria from other mainly anaerobic habitats are regarded as putative syntrophic propionate oxidisers (SPO), closely interacting with methanogens [46]. Similarly, the termite gut Cloacimonetes seem to have the same genomic potential towards SPO oxidation than the other known Cloacimonetes, making it a putative syntrophic partner of methanogenic archaea in soil-feeding termites. The other, previously sequenced termite gut Cloacimonetes was also reconstructed from a soil-feeding *Labiotermes* species [30]. Further comparisons of the genomic content of these two Cloacimonetes MAGs with other Cloacimonetes genomes indicated the enrichment of genes associated with cell mobility in the termite cluster Cloacimonetes (data not shown). Indeed, bacteria in the termite gut system are known to be highly mobile, possibly allowing them to actively reach their preferred substrate or swim against the physico-chemical gradients [9, 47]. The cyanobacterial MAG was affiliated with the Vampirovibrionia group (previously known as Melainabacteria), and to our knowledge, it represents the first MAG of this sister group of Cyanobacteria ever reconstructed from the termite gut microbiome. Nevertheless, its metabolic activity in the termite gut system seems minor, as could be further deduced from the gene expression profiles (see below).

One of the MAGs was affiliated with a previously reconstructed archaeal MAG from a *Labiotermes* species study [30] and assigned to the Methanomethylophilaceae family (Additional file 1: Table S3). Based on the ANI comparison, both archaeal MAGs represented different species. Three other low quality Methanomethylophilaceae MAGs were additionally reconstructed (Additional file 1: Table S2), suggesting that this archaeal family dominates the *Labiotermes* gut.

### Metabolic potential towards lignocellulose digestion of main gut bacteria

In continuation, by mapping metagenomic and metatranscriptomic reads to the reconstructed MAGs, we determined the abundance (averaged over the hindgut compartments, as we only sequenced total hindgut metagenome representing the bacterial luminal fluid content) and activity of specific bacterial groups in the different gut compartments of *L. labralis* workers. In addition to the discussed Cloacimonetes MAG, it was mainly Clostridia and two Treponema (Spirochaetes) that were among the most abundant MAGs in the termite hindgut metagenome. Not surprisingly, most of the MAGs proved to express their genes in the anterior hindgut compartments (Fig. 2B), mainly P3 which was also characterised with the highest bacterial richness and diversity (Fig. 1B). The P3 compartment is considered a preferred niche of microbial activity in the termite digestive tract [1]. Two MAGs assigned to Firmicutes (MAG23 and MAG28), both Clostridia, and one Proteobacteria (MAG2 affiliated with Xanthobacteraceae) were characterised with the highest metatransciptomics abundance in the P1 section of the hindgut. Bacterial MAG33 representative of Cyanobacteria, MAGs 13, 24 and 35 all assigned to Firmicutes, Patescibacteria MAG32 and MAG20 of Proteobacteria origin were characterised with the lowest transcriptional activity across the studied termite digestive tract compartments. We did not manage to link any OTU with a reconstructed MAG.

We further analysed the CAZyme genomic content and gene transcript expression levels in the different gut sections. In total, 1,713 of CAZy domains localised in 1,638 genes were detected in reconstructed good-quality MAGs, and the highest number was assigned with GHs (990 genes), glycosyl transferases (GTs; 404), carbohydrate esterases (CEs; 150) and carbohydrate binding domains (CBMs; 56), followed by pectin lyases (PLs, 23) and auxiliary activity enzymes (AAs; Additional file 1: Table S5). Representatives of Fibrobacteres and Firmicutes were characterised with the highest CAZyme gene coding frequency, 3.4% and 3.1%, respectively. The two phyla also encoded the highest diversity of GH gene families, 16.6 ± 8.4 and 10.5 ± 4, respectively, for Firmicutes and Fibrobacteres MAGs. The numbers are less representative for the latter phylum, as only two MAGs were reconstructed here. The highest number of GH genes was affiliated with GH5 (133 genes), and in particular mainly with a sub-family GH5_4, previously shown to contain multi-functional enzymes of Spirochaetes termite gut origin, with a specificity towards mannan, cellulose and xylan [8]. The most widespread GH family was GH13, and it was detected in 85% of MAGs. This family includes enzymes involved in the degradation of α-glucans (*i.e.* polysaccharides with glucose units linked by α(1 → 4) glycosidic bonds), and it is also widely expressed by the host in the upper part of the digestive tract (see below). The other widely encoded GH families included GH109 (acting on α-N-acetylgalactosamine, *i.e.* bacterial cell wall), GH23 (peptidoglycan and chitin), GH77 (starch) and GH3 (β-glucosidase and xylosidase). Of these, only GH3 and GH77 were defined as present in over 85% of analysed termite species, according to the recent analysis of 129 termite gut metagenomes [7]. Differential utilisations of GH genes by distinct MAGs, as deduced by the gene expression levels (Fig. 2D), further highlights specialisation towards the different lignocellulosic and non-cellulosic polysaccharides. Among carbohydrate esterases, CE4 genes were present in multiple copies in nearly every reconstructed MAG, supporting the bacterial hydrolysis of hemicellulose (*i.e*. different types of xylans), as well as chitin and peptidoglycan. In total, 14 different CBM families were detected, with CBM48 (glycogen binding) being the most widespread. Again, this supported the preference of the termite gut microbiome towards easy-to-degrade α-glucans [8].

The number and diversity of bacterial oxidative enzymes known to be involved in lignin degradation and modification (*i.e*. AA1, 2, 4, etc*.*) was much lower compared to hydrolytic GHs, and no lytic polysaccharide monooxygenases (LPMOs) were detected (*i.e.* AA10; Additional file 1: Table S5). This result strongly contrasts with the observation that the soil-feeding termite gut microbiota is mainly active in the degradation of reduced substrates, including tannin-protein complexes and polyaromatics resulting from lignin degradation [4]. The Proteobacteria-assigned MAG2 encoded the largest repertoire of AAs, with six out of eight AA genes belonging to the AA3 family (mainly AA3_2), representing cellobiose dehydrogenases. Cellobiose dehydrogenases are extracellular enzymes whose function is to reduce the LPMOs via extracellular electron transfer, thus supporting oxidative polysaccharide depolymerisation [48]. Currently, AA3 genes in the CAZy database are exclusively of fungal origin, with just one putative AA3 gene sequence being of prokaryotic origin, namely a halophilic archaeon of the *Halorubrum* genus [40]*.* Nevertheless, prokaryotic genes assigned as AA3_2 were also present in the metagenomic reconstructions from higher and lower termite guts, as recently reported by [7]. In general, MAG2 was the most transcriptionally active in the highly alkaline P1 compartment, including the highest expression of AA3 coding genes. No LPMOs gene transcripts were detected in any bacterial genome, therefore the function of prokaryotic AA3_2 CAZymes remains speculative.

As a complement, we also verified whether CAZymes tend to cluster in the genomes of the soil-feeding termite gut microbes as previously shown for a grass-feeding *Cortaritermes* species [8]. As a result, we detected 51 putative CAZyme clusters, containing at least one CAZyme, one transporter and one transcriptional regulator (Additional file 1: Table S6). As expected with the overdominance of Firmicutes, these clusters were mainly detected in the reconstructed clostridial genomes, and only two clusters were from Cloacimonetes and Spirochaetes origin. GH2, GH4, GH9 and GH94 were the most frequently clustered CAZymes. Due to a high level of MAGs fragmentation, we suspect that the real number of CAZyme clusters is much larger, and clustering CAZymes into functionally complementary units is a general trend in bacterial genomes from biomass-rich habitats (own data, unpublished).

### *De novo* metatranscriptomics and termite host gut transcriptomics

To further understand the lignocellulose degradation mechanisms in the different termite gut compartments, we separately reconstructed the de novo termite gut metatranscriptome and host gut transcriptome. The de novo co-assembly of reads from the six termite gut samples resulted in 2,090,052 contigs, with 2,114,234 recognised ORFs, corresponding to partial and complete gene transcripts. Of these, roughly 30% were taxonomically annotated. Gene transcripts of bacterial origin corresponded to 369,503, representing around 17.5% of recovered ORFs. In accordance with the gut microbial community structure, most of the gene transcripts were of Firmicutes origin, mainly Clostridia and to a lesser extent Bacilli, followed by Spirochaetes, Bacteroidetes and Actinobacteria (Additional file 2: Fig. S1). The highest transcriptional abundance of Firmicutes-assigned gene transcripts was in the anterior hindgut part (P1 and P3), while Actinobacteria, Bacteroidetes and Proteobacteria dominated the posterior hindgut compartments. Firmicutes and Actinobacteria were also the most transcriptionally active phyla in the foregut, while the highest transcriptional abundance of Spirocheates-assigned gene transcripts was recorded in the midgut. Overall, metatranscriptomic profiles correlated well with the abundance of specific phyla in the different gut compartments (Fig. 1D, Additional file 2: Fig. S1). Archaea gene transcripts were very low abundant and mainly present in the posterior hindgut as well as in the P4 compartment, and were dominated by Euryarchaeota.

**Fig. 3:**
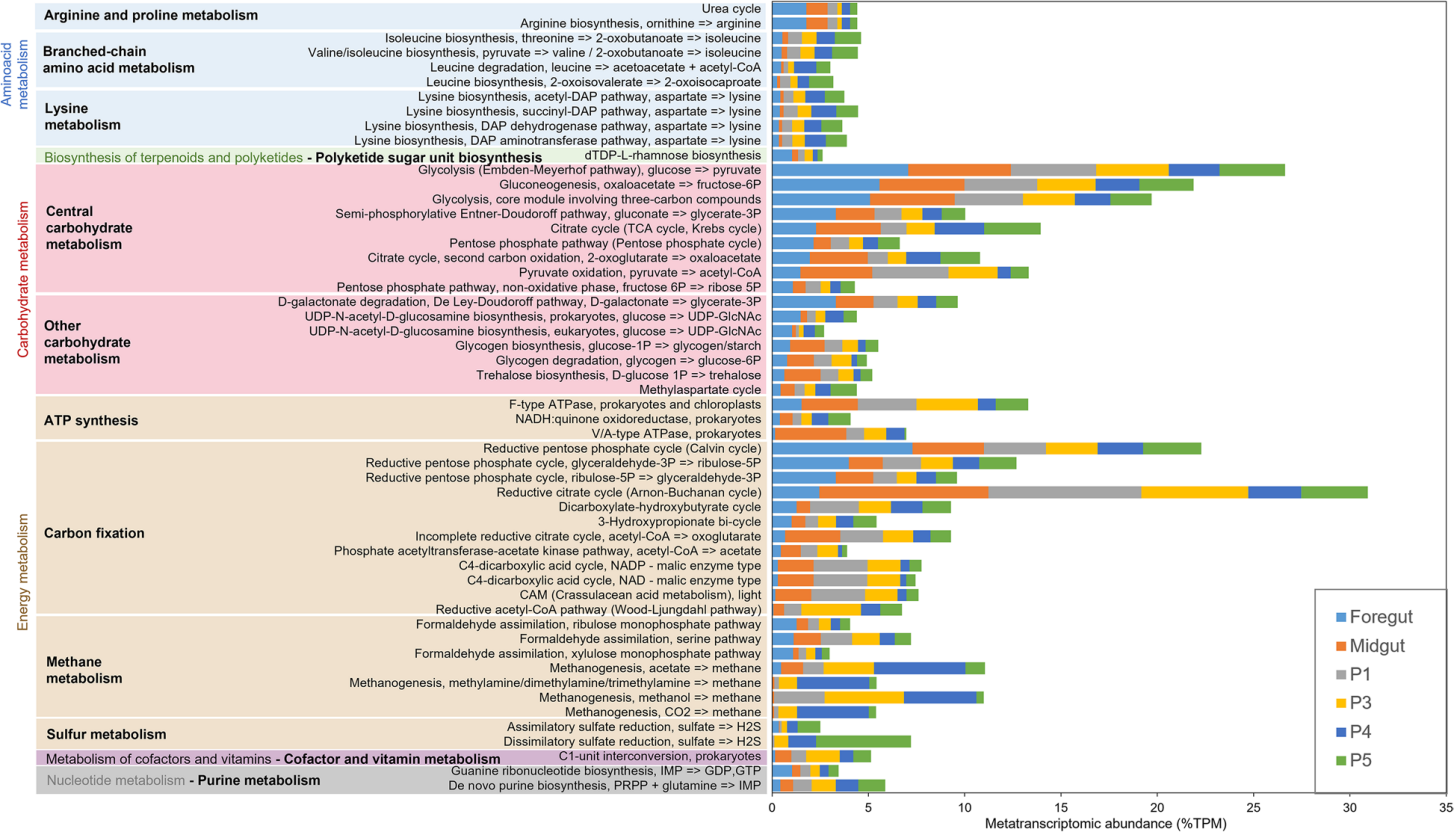
Main metabolism pathways employed by the termite gut bacteria in the different compartments of the digestive tract and their metatranscriptomic abundance

Community-wise, metatranscriptomic profiles relative to the different gut compartments were dominated by distinct functional groups (*i.e.* KOs), indicating different microbial activities in the different gut sections (Additional file 1: Table S7). Flagellin was overall the most transcriptionally abundant KO in the midgut and anterior hindgut only, suggesting that mainly bacteria residing in these gut compartments are motile. This characteristic was largely reduced in the posterior hindgut and was nearly absent from the foregut. As expected, and also in line with previous reports [16], methanogenesis was dominant in the posterior hindgut, where the pH is closer to the neutral values. Interestingly, acetoclastic methanogenesis prevailed in P4 and less in P3, and hydrogenotrophic mainly in P4, while methanogenesis from methanol also took place in P1, in addition to P3 and P4 (Fig. 3). Sulphate reduction seemed to take place mainly in the last hindgut compartment, characterised with the lowest pH. A relative abundance of gene transcripts associated with the urea cycle was highest in the foregut and midgut. Gene transcripts assigned to the KOs relative to carbohydrate hydrolytic enzymes (*i.e.* EC:3.2._) accounted for 1.33% ± 0.34 of all functionally assigned gene transcripts. This number is only slightly lower than the carbohydrate hydrolytic KOs that accounted for 1.99% ± 0.27 of all functionally annotated genes for a grass-feeding *Cortaritermes* species [8]. This indicates that the soil-feeding *Labiotermes* still feeds largely on cellulosic and non-cellulosic polysaccharides present in soil. Nevertheless, to complement the presumably low-carbon diet, microbes in the *Labiotermes* gut system actively fix CO_2_ employing distinct autotrophic carbon fixation pathways in the different compartments (Fig. 3, Additional file 1: Table S8). Photosynthetic Calvin cycles dominate in the foregut, most probably as a residual activity of ingested algae, cyanobacteria and other photosynthetic bacteria present in soil. A reductive citric acid cycle (Arnon-Buchanan cycle) prevails in the midgut and anterior hindgut, while the Wood-Ljungdahl pathway seems to be less employed by bacteria, and low-abundant gene transcripts are only present in P1-P4 compartments.

Similar to the metatranscriptome assembly, six separate termite host gut libraries were de novo co-assembled into 218,010 contigs and of the predicted ORFs 14,281 were assigned to Insecta origin. The completeness of the termite gut transcriptome was assessed at 71.5%. A functional annotation and abundance of the termite de novo reconstructed gene transcripts is given in Additional file 1: Table S9. Briefly, the enzymes involved in peptide hydrolysis (*i.e.* EC:3.4._) were among the most highly expressed metabolic genes in the foregut and midgut sections, with a trypsin KO1312 being the most expressed functional gene category in the midgut. A high abundance of peptidases-encoding gene transcripts suggests that the host metabolism is centred at protein degradation. Bacterial biomass is suggested to be the major source of proteinaceous food [49, 50]. Chitinase (KO1183), endoglucanase (KO1179) and α-glucosidase (KO1187)-coding gene transcripts were among the most abundant carbohydrate hydrolases in the midgut compartment. Further analysis of the termite gut transcriptome is limited to the host carbohydrate metabolism (see below).

### Bacterial lignocellulolytic activities in *Labiotermes* gut compartments

**Fig. 4:**
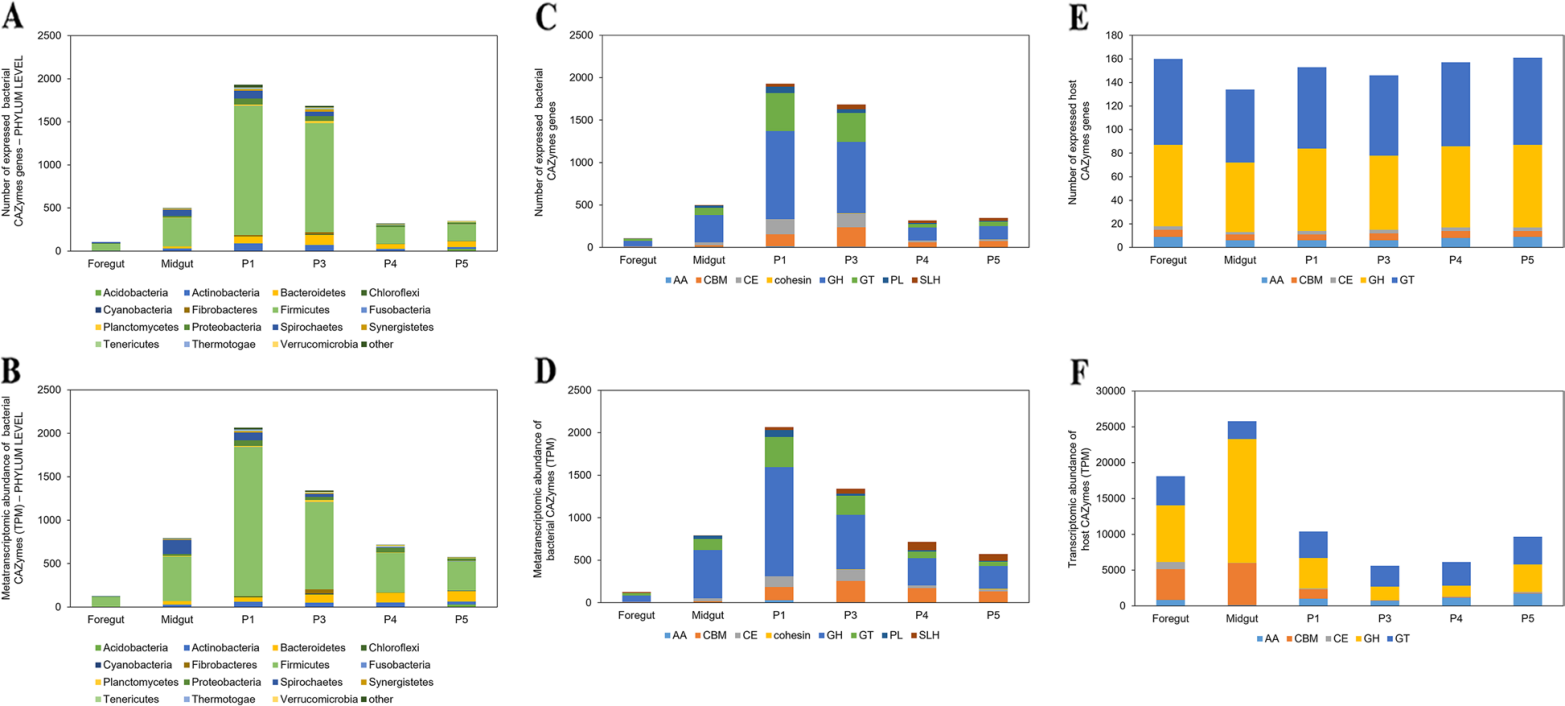
Characterisation of the lignocellulolytic metabolism of the host and its microbiome in the different gut compartments, according to the de novo metatranscriptomic reconstruction. **A** Number of expressed microbial carbohydrate active enzyme (CAZyme) coding genes and their metatranscriptomic abundance (**B**), according to the phylum classification. **C** Number of expressed microbial CAZymes and their metatranscriptomic abundance (**D**), according to the CAZy class assignment. **E** Number of expressed host CAZymes and their transcriptomic abundance (**F**), according to the CAZy class assignment

An analysis of the reconstructed bacterial community metatranscriptome allowed for the identification of 3,534 CAZymes-domains of exclusively bacterial origin (Additional file 1: Table S10), of which roughly 197 were also reconstructed in bacterial MAGs of Firmicutes, Fibrobacteres, Spirochaetes and Proteobacteria origin mainly. In accordance with a previous report, only a low overlap between the termite gut metagenome and metatranscriptome could be achieved [8], highlighting the importance of the metatranscriptomic reconstruction to obtain a complete picture of the termite gut microbiome lignocellulolytic capacities. In total, 166 different main CAZyme families were detected, or 239 if counting sub-families, which is significantly higher than the recently reported numbers for other soil-feeding termite gut metagenomes, on average 77.2 ± 42 CAZyme categories [7]. The high diversity of microbial CAZyome may be linked to the fact that the *Labiotermes* considered a true soil feeder consumes the poorest quality biomass, and to achieve an acceptable nourishment level it must employ a larger portfolio of digestive strategies (*i.e.* a higher diversity of CAZymes) to efficiently assimilate the whole variety of nutritional resources available in soil. This could contrast with the lower CAZyme diversity in termites occupying the wood/soil interface (often categorised as soil feeders), which are associated with tree root systems, or highly humified but still recognisable organic litter [4]. The de novo reconstructed CAZymes in *L. labralis* metatranscriptome included GHs with 1,792 assigned gene transcripts, GTs (768), 15 AAs genes, CBMs containing genes (400), CEs (318), and to a much lesser extent, PLs (107) and SLHs (128). The highest diversity of bacterial CAZyme gene transcripts were of Firmicutes (2577 genes), Bacteroidetes (274), Actinobacteria (161), Proteobacteria (143), and Spirochaetes (136) origin, presumably making some of the members of these phyla dominant lignocellulose hydrolysers in the termite gut system studied. CAZyme gene transcripts affiliated with the above bacterial phyla were detected in all of the studied gut compartments (Fig. 4A and B). However, the highest metatranscriptomic abundance and the largest number of expressed genes were in the anterior hindgut, which is typically the main site of bacterial presence and activity in the termite gut [1]. Anterior hindgut is also the environment of the highest lignocellulolytic activity of Firmicutes, while Bacteroidetes seem to be more actively involved in polysaccharide degradation in the posterior hindgut. Spirochaetes are the most transcriptionally active in the midgut and P1, while CAZyme gene transcripts of Actinobacteria and Proteobacteria were equally abundant across the whole gut system, with the exception of the foregut. Verrucomicrobia, which were relatively abundant in the posterior hindgut based on the 16S rRNA gene amplicon analysis (Fig. 1D), were slightly more active in P4 and P5, although the overall gene transcripts abundance was lower compared to the other phyla. Fibrobacteres, which next to Spirochaetes are the main biomass hydrolyser in the wood- and grass-feeding higher termites [8, 9], expressed some of their lignocellulolytic potential mainly in the P3 hindgut compartment (Fig. 4B).

**Fig. 5:**
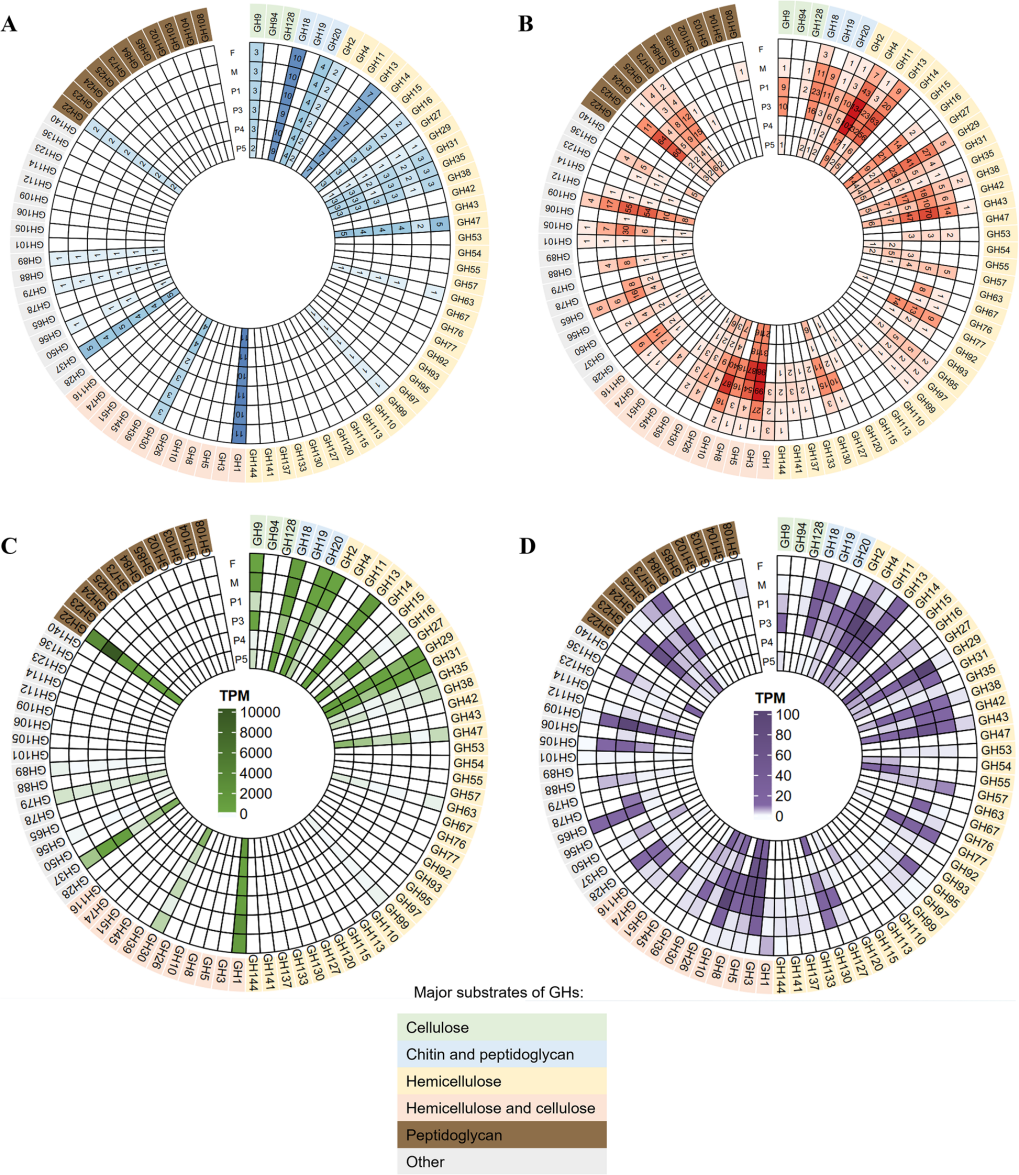
Characterisation of glycoside hydrolases (GH) in the termite gut metatranscriptome and host transcriptome. Number of GH gene transcripts identified across distinct gut compartments in the host gut transcriptome (**A**) and in the termite gut symbionts metatranscriptome (**B**). GH expression profiles across distinct gut compartments in the host transcriptome (**C**) and bacterial metatranscriptomes (**D**)

Glycoside hydrolase was the most abundant enzyme class, and the diversity as well as the transcriptional abundance of expressed genes was highest in the anterior hindgut (Fig. 5). In total, 75 different GH families were detected and most of the annotated gene transcripts were of Firmicutes and Bacteroidetes origin. The largest diversity of genes was assigned to GH4 (232 gene transcripts), followed by GH3 (198), GH5 (140) and GH10 (118; Additional file 1: Table S10). Evidently, there is a niche specialisation, and the different bacterial players utilise different GHs to target specific lignocellulose fibres in the different gut compartments. In the foregut part, α-glucans targeting GH13 enzymes are employed by Firmicutes to degrade starch and trehalose. Further degradation of starches (GH4) and fucose (GH29) was the main activity taking place in the midgut, and the most abundant gene transcripts were of Firmicutes (GH29 and GH4) and Spirochaetes origin (GH29). The xylan-targeting endo-acting enzymes GH10 and GH11 are mainly employed by Firmicutes in P1 and to a lower extent in P3 gut parts, which are the most highly alkaline compartments [44]. Collectively, these two GH families were also among the most transcriptionally abundant GHs, showing the importance of hemicellulose in the diet of the soil-feeding *Labiotermes*. Of the main cellulose targeting GH families (*i.e*. GH1, GH3, GH5, GH9), GH3 and GH5 gene transcripts from Firmicutes were the most abundant in P1-P4. Actinobacteria seem to employ their GH3 and GH5 genes across most of the termite gut system, however, different genes are expressed in the different gut compartments. Bacteroidetes are mainly actively involved in cellulose degradation in the posterior hindgut, with much less activity in P1 and P3. Interestingly, while GH3 enzymes are rather compartment-specific, genes encoding GH5, and in particular GH5_4, are expressed over the different hindgut compartments, characterised with distinct pH values.

Bacterial contribution to the oxidative lignocellulose degradation was highest in P1, and most of the highly expressed AA gene transcripts were assigned to Firmicutes, mainly AA4, AA7 and AA10 families. The latter contains known bacterial LPMOs. Only five AA6 gene transcripts were detected, three of Firmicutes origin, and one each assigned to Proteobacteria and Deinococcota, all of them mainly expressed in the anterior hindgut compartments. Most of the bacterial CE genes were also expressed in the anterior hindgut, with the highest gene transcript abundance being measured in P3 (Fig. 4C, D). The dominant CE family in terms of the number of reconstructed gene transcripts and the overall transcript abundance was CE4 (191 gene transcripts), commonly involved in the deacetylation of xylan and chitin. CE1 was the second most abundant (38 gene transcripts) and highly expressed family. It contains known feruloyl esterases, *i.e.* enzymes which break the linkages between the lignin and hemicellulose moieties, and it is mainly bacteria of Firmicutes origin that employ this enzyme in the different hindgut compartments.

### Diversity and expression patterns of host CAZymes

To complement the prokaryotic lignocellulolytic activity in the termite gut system, we also analysed the host CAZyome in the different gut compartments. As a result, a search for CAZyme gene transcripts in the reconstructed host transcriptome revealed the presence of 184 CAZymes assigned to AAs (4 families), CBMs (4), CEs (3), GHs (22), and GTs (36), with the latter two classes comprising the majority of the expressed genes (Fig. 4E, F; Additional file 1: Table S11). In comparison with the termite gut metatranscriptome, most of the host CAZyme coding genes were simultaneously expressed in the different gut compartments, however, the highest transcriptomic abundance was present in the foregut and midgut, and the lowest in P3, where most of the microbial activity takes place. This remains in agreement with previous molecular and enzymatic reports [1, 51], and designates anterior compartments of the termite digestive tract as the main sites of the activity of the host hydrolytic enzymes, excreted by the labial gland and the midgut epithelium, respectively. Interestingly, presumed activity of the same enzyme in the different gut compartments, characterised with a very distinct pH, would indicate a termite host itself as a source of CAZymes with an unusually high pH tolerance.

The largest discrepancy between the host and prokaryotic CAZyomes was a proportionally higher transcriptional abundance of genes hosting the CBM domains in the host transcriptome, specifically those assigned to the CBM13 and to a lesser extent, to CBM14 families. In total, 22 different GH families were identified in the termite transcriptome, suggesting a relatively broad hydrolytic potential of the host. The most highly expressed gene family was GH22, which encodes for lysozyme, and the highest transcript abundance was recorded in the midgut followed by the foregut (Fig. 5). As in the case of other soil-feeding insects (*e.g.* the Scarabaeidae beetle; [52]) and soil-feeding termites [7], bacterial biomass present in the soil matrix is hydrolysed, providing essential nutrients to the termite host. In addition, lysozymes excreted from the insect upper digestive tract, including the salivary glands, play a role in immune response [50]. Gene transcripts affiliated with plant-biomass targeting CAZyme families GH1, GH13 and GH18 were mainly expressed in the foregut and midgut sections. The highest transcriptomic abundance of the three GH9 gene paralogs encoding for an endoglucanase was in the midgut and is in agreement with previous reports on the concentration of the β-1,4-endoglucanase activity in the midgut of wood-feeding termites [53]. While many GH families seem to be expressed by both the termite host and the gut microbiome, some were restricted to the host only, and these included GH37, GH47, GH56, GH63, GH79, GH89, and GH152 (Fig. 5). In turn, the number of GH families exclusively present in the gut metatranscriptome was much higher, and included GH3, GH4, GH5, GH8, GH10, GH11, GH23, GH29, GH43, and GH109 among the most transcriptionally abundant.

### Comparison of termite endogenous CAZymes across different termite species

**Fig. 6:**
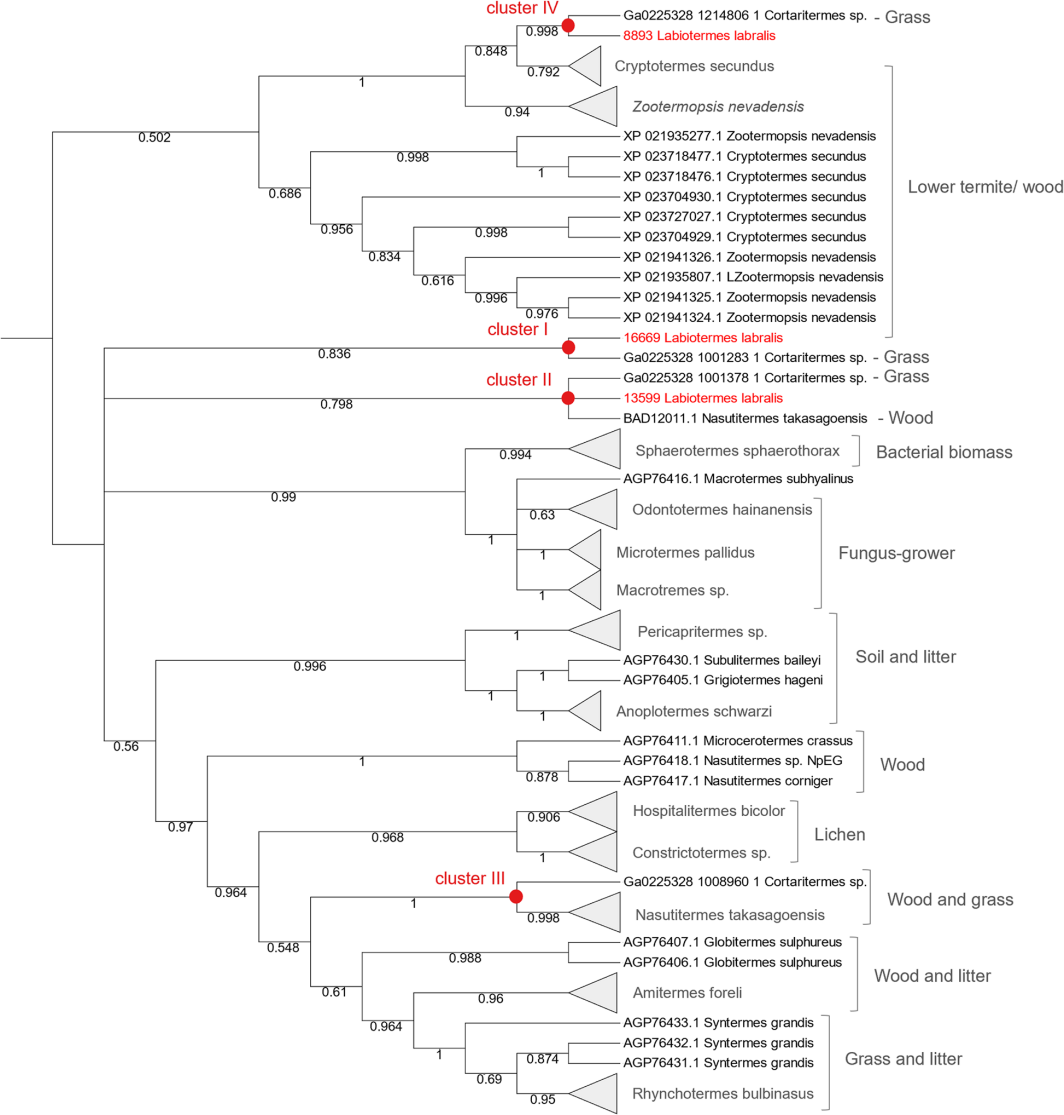
Rooted neighbour-joining tree of the GH9 termite endoglucanases. Percentage of replicate trees, in which the associated taxa clustered together in the bootstrap test (500 replicates) are shown next to the branches. The evolutionary distances were computed using the Poisson correction method and are in the units of the number of amino acid substitutions per site. Due to the presence of partial GH9 protein sequences, this analysis involved only 69 amino acid sequences of the 462 total positions in the final alignment

In continuation, we compared the CAZyome of *L. labralis* to that of *Cortaritermes* sp. ([8]; both corresponding to the de novo reconstructed transcriptome) as well as to the CAZymes identified in *Macrotermes natalensis* [54], *Zootermopsis nevadensis* [55] and *Cryptotermes secundus* genomes (GenBank assembly accession GCA_002891405.2). As a result, the overall diversity patterns of the CAZyme families detected were similar among all the termite species, despite their completely different feeding regimes (Additional file 1: Table S12). In general, the cellulolytic activity is expected to be weaker in soil-feeding termites than in the wood feeders, therefore we further investigated the evolution of the termite GH9 cellulases to better understand the ability of *L. labralis* to feed on different forms of cellulose present in soil. To infer the enzyme phylogeny, we incorporated the higher termite endoglucanase sequences from [56] that were supplemented with (partially reconstructed) GH9 proteins from *L. labralis* and other termite species including lower termites (Fig. 6). For most of the termite species, GH9 endoglucanase paralogs from the same species clustered together in species-specific clusters, confirming previous observations [56]. The only exceptions were the soil-feeding *L. labralis*, the wood-feeding *Nasutitermes takasagoensis* and the grass-feeding *Cortaritermes* sp. whose endoglucanase paralogs co-clustered together, regardless of the species phylogenetic distance and different diets. Three of these clusters grouped together with the soil- and wood-feeding higher termites (two of the three co-clustered with *N. takasagoensis*; clusters I-III, Fig. 6). The fourth cluster contained *L. labralis* and *Cortaritermes* sp. enzyme sequences, which were largely similar to endoglucanases of phylogenetically basal lower termites (96 to 98% of identity at the protein level) and were grouped separately from higher termites. The endoglucanases of all the other soil-feeding termites investigated here, including *Anoplotermes*, *Grigiotermes*, *Subulitermes* and *Pericapritermes* genera, co-clustered together in species-specific groups, forming one soil and litter feeding termite cluster (Fig. 6; [56]).

Highly similar paralogous endoglucanases of the same species most possibly arose through gene duplication, as previously suggested for other termites [56]. A higher sequence homology likely imposes a similar specific activity on the produced enzyme, restricting the cellulolytic phenotype of the host. By contrast, the structurally distinct paralogous endoglucanases of *L. labralis*, *Cortaritermes* sp. and *N*. *takasagoensis* possibly show a different specificity towards distinct forms of cellulose. Such a concept has not yet been tested for termite enzymes, however, it was recently documented for cellulases from the *Cellulomonas fimi* bacterium [57]. The authors showed for example that the difference in the cellulose processivity of two GH6 cellulases was presumably due to their structural difference. In this case, processivity was defined as an extent of a continuous hydrolysis without enzyme detachment from the substrate, allowing for an efficient degradation of crystalline cellulose, for example. Such a capacity could render some termite species more efficient than others in cellulose digestion. This could possibly contribute to the ecological success of *Labiotermes* sp. which is one of the most abundant species in the Amazonian rainforest [14]. Further studies at the genomic level of the different termite host CAZyomes could shed light on the underlying genetic principles of the termite dietary evolution.

### Complementary contribution of the host and its gut microbiome to soil polysaccharide degradation

Based on the variety of prokaryotic CBM domains, which is significantly larger than for the termite host, the gut microbes can target a much more diverse range of polysaccharides present in soil. Briefly, host enzymes would primarily target α-glucans (*i.e*. starch; CBM13, CBM21) and chitin (CBM14), with the majority of CBM-carrying genes being expressed in the foregut, midgut and P1. Increased host lignocellulolytic activity in P1 may actually be attributed to the mixed segment, which was not specifically targeted in our study. No cellulose-targeting termite CBM genes were discovered, even though at least three GH9 paralogous endoglucanase genes are encoded in the *L. labralis* genome. The expression of specific genes carrying prokaryotic CBM domains seems to be compartment-specific, which is in accordance with a previously published metatranscriptomic analysis of the gut compartments of the wood-feeding *Coptotermes formosanus* lower termite [51]. The most widely expressed prokaryotic CBMs were also characterised with the highest transcriptional abundance. They included xylan targeting CBM9, CBM54, CBM4 and CBM22 as well as galactose, mannose and, alternatively, lactose targeting CBM13 and CBM32 and peptidoglycan and chitin binding CBM50.

The enzymatic degradation of soil organic matter which contains diverse polysaccharides, including lignocellulose components and recalcitrant chitin and peptidoglycan, both co-polymerised with polyphenols, starts in the foregut and continues across the whole gut system with the sequential detachment and removal of lignin, hemicellulose, and cellulose particles, as well as an intensive degradation of bacterial biomass. Based on the gene expression profiles, the host enzymatic machinery active in the upper digestive tract would first extract freely available simple sugars and oligo/disaccharides, *e.g*. glucose, mannose and mannooligosaccharides, galactose, cellobiose, etc. α-glucans would also be preferentially degraded in the foregut. For example, the gene transcript abundance of GH13 showing α-amylase activity is highest in this compartment (Fig. 5). Some microbes also benefit from the relatively easily accessible non-cellulosic polysaccharides and simple sugars already in the foregut and midgut by expressing for example their α -amylases (GH13), β-glucosidase (*e.g.* GH1, GH2, GH3, GH5, etc*.*) or maltose phosphorylases (*e.g.* GH65). Based on the CAZyme expression profiles, the upper digestive tract is also the site of bacterial biomass and chitin degradation, which confirms earlier reports [58]. Host lysozyme activity is expressed throughout the whole digestive tube, but GH22 gene transcripts are most abundant in the midgut and foregut compartments. Expression of the host lysozyme activity in the different gut compartments indicates that termites may simultaneously depend on their gut microbiota as mutualists and exploit them as a food source. In addition to providing essential nutrients to the host [52], a high bacterial biomass turnover could serve other purposes. First, the enzymes released from hydrolysing microbes present in soil or upper gut compartments, constitute a pool of “public goods” helping other microbes to degrade biomass faster [59]. Second, the degradation of bacterial biomass that is carried over from upper gut compartments together with the digested food, would minimise the competition between the microbial populations present in the different gut sections, indirectly promoting the different gut compartments to specialise in the efficient utilisation of separate biomass fractions. Third, a significantly higher expression of the host lysozyme coding genes in the upper digestive tract could also be a way to control the microbial population present in the foregut and the midgut, in order to assure the maximum utilisation of nutrients by the host. Hindgut bacteria also actively express lysozymes (*i.e.* GH23-GH25, and GH73), and the highest transcriptional abundance was observed in the P1 and P3 compartments.

Chitin is the second most abundant polymer in nature, also widely present in a tropical soil where it originates mainly from the decomposition of the exoskeletons of arthropods and fungi. Constantly complementing the diet with chitin, which is also a source of nitrogen, was recently suggested for the grass-feeding higher termite *Cortaritermes* sp., based on high gene expression levels of chitin-targeting enzymes [8]. *Labiotermes* seems to utilise chitin through a combined action of GH18 (chitinase activity prevails among the characterised eukaryotic GH18 enzymes) and CE4 (chitin deacetalyse is the only activity of the characterised eukaryotic CE4 enzymes described). Gene expression is most prominent in the foregut and midgut compartments and continues across the whole gut system. Bacterial chitin degradation also starts in the midgut but chitinases and chitin deacetylases are mainly expressed in the anterior hindgut. Chitin degradation by the lower termite gut flagellates was recently proposed to inhibit infection from entomopathogenic fungi [60]. A similar function could potentially be ascribed to gut prokaryotes in higher termites, but this would require further investigation.

On average, lignocellulose is composed of cellulose (40—50%), hemicellulose (25—30%) and lignin (20—25%), with a smaller amount of pectin (5—10%). Lignin degradation in termites still remains an open question, and except for the fungus-growing termites, lignin is considered the major constituent of termite faeces [61, 62]. Early studies of soil-feeding termites reported low levels of enzymatic activity towards lignin deconstruction in the intestines of *Cubitermes*, *Noditermes*, and *Procubitermes* [63]. The lignin-targeting capacity of *L. labralis* has not yet been assessed, but according to the transcriptomic profiles, the host could express its own lignin-targeting enzymes across the whole intestinal tract. First, laccases of the AA1, which are mainly expressed in the foregut, would start depolymerising lignin allowing cellulases and microbial hemicellulases to access the other lignocellulose fractions in the hindgut more easily. Second, vanillyl alcohol oxidases of AA4 would continue the degradation of specific lignin components in P4, suggesting that the humified derivatives of lignin (*i.e.* aromatic substrates), could be further degraded to the nutritional benefit of the host. Both enzyme types operate in compartments characterised with a largely neutral pH and a relatively high partial pressure of oxygen, in line with the principle of known lignin degradation pathways [64]. Bacterial contribution to lignin degradation seems very limited in the *L. labralis* gut, and one AA1 gene and five AA4s were detected in the de novo reconstructed metatranscriptome, all characterised with a relatively low transcriptional abundance. In general, only a limited number of bacteria are currently known to degrade lignin, and these include representatives of Actinomycetes and Proteobacteria [64], which are characterised by low abundance in the *Labiotermes* gut.

Host cellulose degradation takes place across the whole termite gut, but the maximum gene expression is present in the midgut, and the main activity is attributed to GH9, with the highest transcript abundance coming from the Labio_GH9 cluster I (Fig. 6). Cellobiose dehydrogenase (AA3) combined with the LPMO activity (AA15) contributes to the cellobiose degradation to glucose moieties across the whole gut, but surprisingly, its maximum expression takes place in P5. Possibly, once lignin and hemicellulose fibres are dissociated, residual cellulose moieties can be subsequently accessed by the host. These expression profiles of cellulose-targeting enzymes correlate with the expression of β-glucosidase (*i.e.* mainly GH1 and GH30), which is highest in the midgut and in P5. Roughly three bacterial LPMOs of the AA10 family were identified in the metatranscriptome and were mainly expressed in the anterior hindgut. The expression of bacterial GHs known to express mainly a cellulolytic activity (*i.e.* GH9 and GH94) was generally low, compared to other CAZymes. However, GH5 and GH10 enzyme coding genes were widely expressed, mainly in the hindgut, with known activities including endoglucanase and endoxylanase [8]. Another widely expressed gene family in P1 was GH11, known to target xylan [65]. Similar to cellulose degradation by the host, cellulose and xylan depolymerisation by bacteria was accompanied by the expression of respective β-glucosidases (*i.e.* GH1, GH3, GH39), and β-xylosidases (*i.e.* GH3, GH43 and GH39), indicating efficient cellulose and xylan utilisation by the *Labiotermes* gut microbes.

## Conclusions

Previously, dual host-symbiont transcriptome sequencing assays were applied to the lower termites *Reticulitermes flavipes* [66] and *Coptotermes formosanus* [51], and a few higher termites including the grass-feeding *Cortaritermes* sp. [8] and a fungus-growing *Macrotermes natalensis* [54]*.* They evidenced a complementary contribution of the host-microbiome to lignocellulose degradation. Here, a similar approach was applied to the higher soil-feeding termite *L. labralis*, providing strong evidence for lignocellulose degradation in addition to bacterial biomass, which is the preferred carbon and nitrogen source of soil-feeders. A high diversity of microbial gene transcript encoding for cellulose and especially hemicellulose targeting enzymes was detected in the different gut compartments. Typically, gene expression was compartment-specific, showing a niche specialisation of specific microbial populations, and the functional adaptation of enzymes to actively process the different lignocellulose components under distinct pH and redox conditions. Indeed, the axial and radial gradients of various physical and chemical parameters are very pronounced between the different gut compartments of soil feeders, compared to other termite feeding groups [15, 16]. From a biotechnological perspective, this makes the *L. labralis* gut system a unique reservoir of carbohydrate-active enzymes showing distinct pH tolerance, which is of high industrial relevance. Compared to microbial CAZymes, the highest transcriptional abundance of host CAZymes takes place in the midgut. However, the majority of host cellulases and other hydrolytic enzymes are also expressed in the other gut compartments, suggesting the existence of a wide range of pH tolerant enzymes. Moreover, alike *Cortaritermes* sp. and *N*. *takasagoensis*, *L. labralis* encodes structurally different paralogous endoglucanases, possibly acquired from different sources during the evolution. This in turn, might indicate their different specificity towards distinct forms of cellulose, rendering this termite species very efficient in cellulose digestion. Currently, none of the *L. labralis* cellulases have yet been biochemically tested, and future studies should shed more light on these unique enzymes.

## Supplementary Information

**Additional file 1.**

**Additional file 2.**

## Data Availability

The raw sequencing reads related to this study are available in NCBI under the following accession numbers: 16S rRNA gene amplicon sequencing reads—PRJNA633772; The metagenomic sequencing reads—PRJNA635664; Host transcriptome sequencing reads—PRJNA634022; Prokaryotic transcriptomes sequencings reads—PRJNA633837. Assemblies and reconstructed MAGS are available upon request.
