# Supplementary material for: A holobiont approach towards polysaccharide degradation by the highly compartmentalised gut system of the soil-feeding higher termite Labiotermes labralis
Source: BMC Genomics. 2023 Mar 15;24:115. doi: 10.1186/s12864-023-09224-5 (PMC10018900; doi:10.1186/s12864-023-09224-5)
Supplement: Supplementary file 2 — Additional file 2. [file 12864_2023_9224_MOESM2_ESM.docx]

Additional File 2:


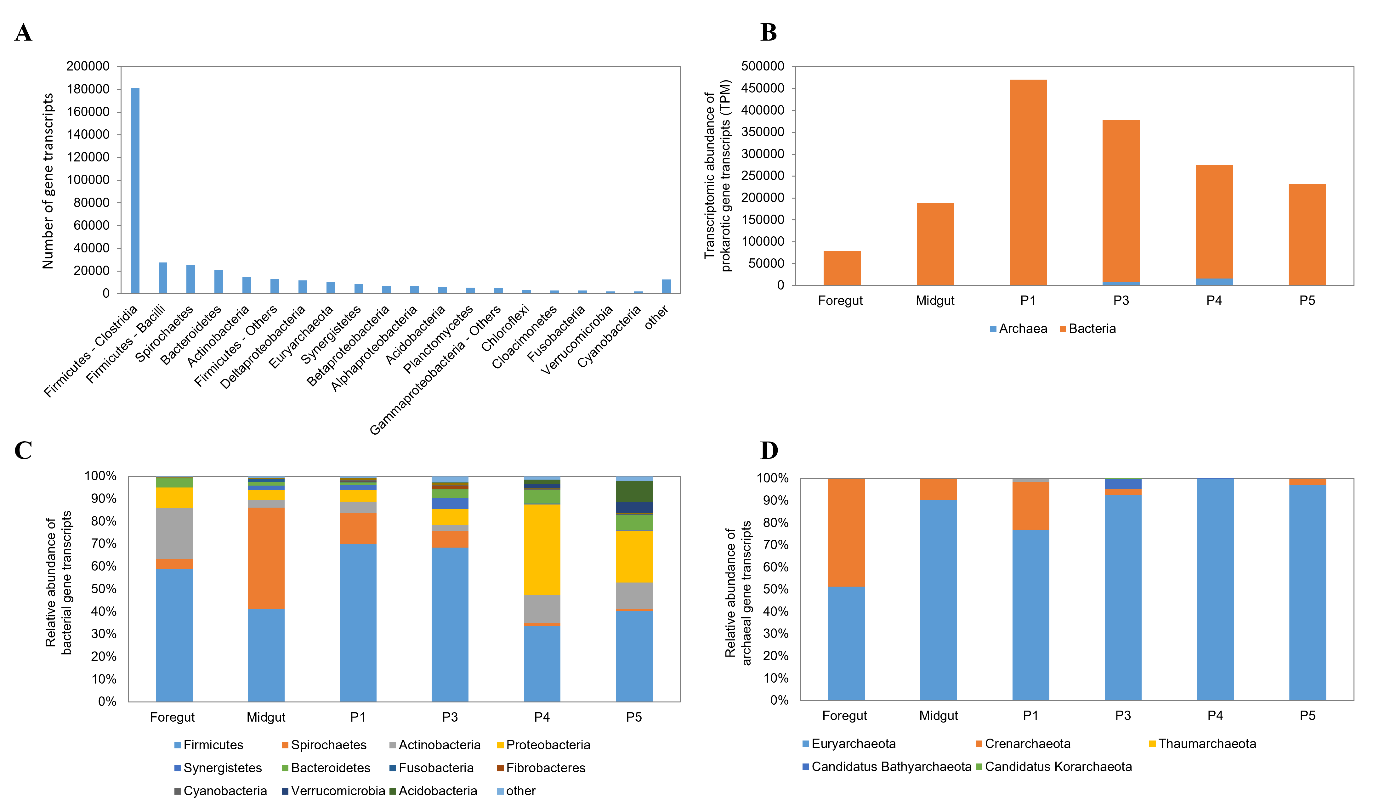


**Figure S1:** Characterisation of the termite gut microbiome. (A) Taxonomic affiliation of prokaryotic gene transcripts. (B) Cumulative abundance of prokaryotic gene transcripts (TMP) in the different gut compartments. (C) Relative abundance of bacterial gene transcripts (%) at the phylum level and in the different gut compartments. (D) Relative abundance of archaeal gene transcripts (%) at the phylum level and in the different gut compartments.


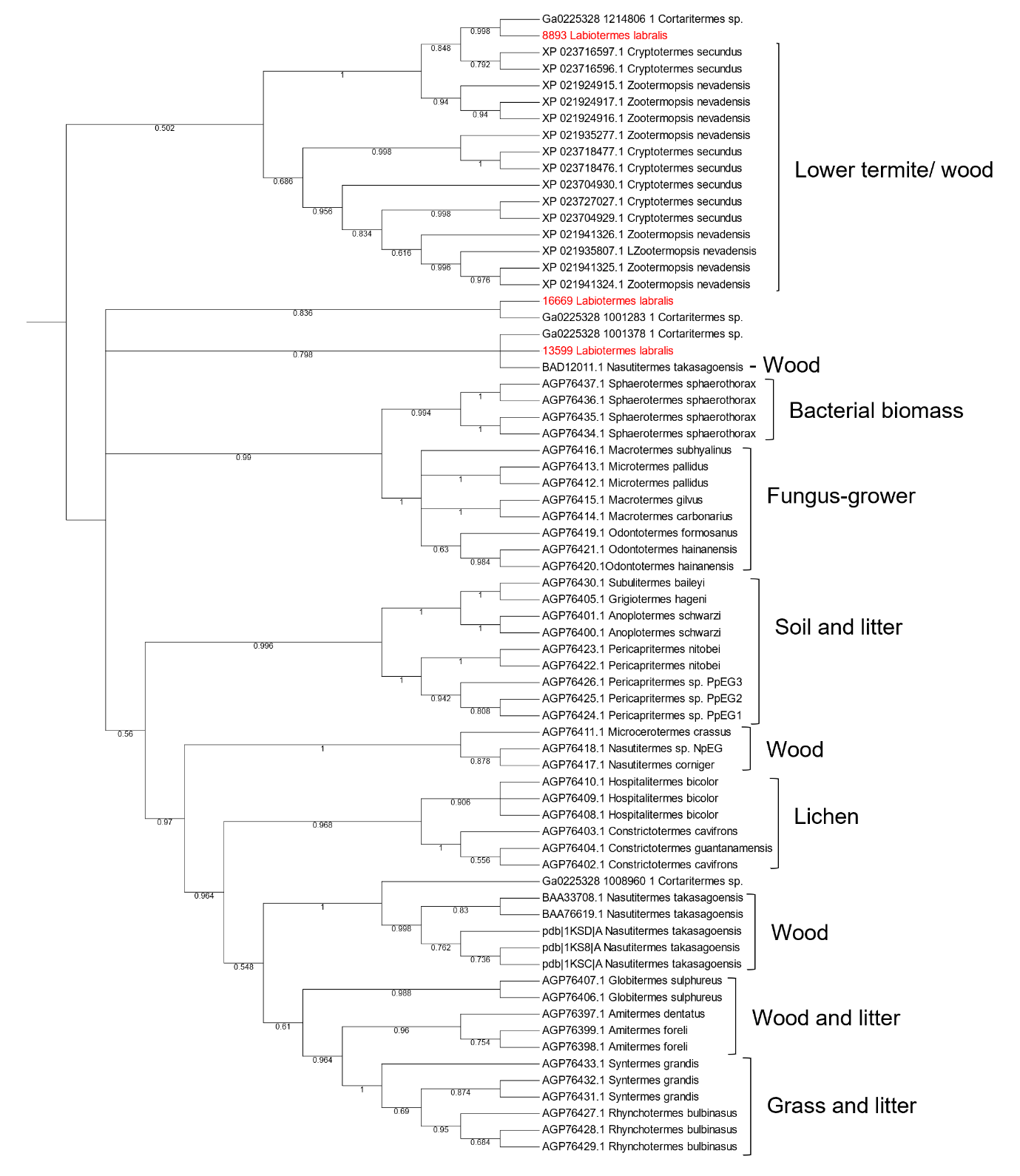


**Figure S2:** Rooted neighbour-joining tree of the GH9 termite endoglucanases. The percentage of replicate trees in which the associated taxa clustered together in the bootstrap test (500 replicates) are shown next to the branches. The evolutionary distances were computed using the Poisson correction method and are in the units of the number of amino acid substitutions per site. Due to the presence of partial GH9 protein sequences, this analysis only involved 69 amino acid sequences of the total 462 positions in the final alignment. Extended version of Fig. 6.
